# What's next for wellbeing science? Moving from the Anthropocene to the Symbiocene

**DOI:** 10.3389/fpsyg.2023.1087078

**Published:** 2023-02-17

**Authors:** Jessica Mead, Katie Gibbs, Zoe Fisher, Andrew Haddon Kemp

**Affiliations:** ^1^School of Psychology, Faculty of Medicine, Health and Life Science, Swansea University, Swansea, United Kingdom; ^2^Regional Neuropsychology and Community Brain Injury Service, Morriston Hospital, Swansea, United Kingdom; ^3^Health and Wellbeing Academy, Faculty of Medicine, Health and Life Science, Swansea University, Swansea, United Kingdom

**Keywords:** Symbiocene, wellbeing, climate action, human suffering, connectedness, sustainability, systems approach

The modern world is now living through the Anthropocene (Slaughter, 2012); a “new human” era signifying the impact that human activities have had on the ecosystems within which we live, characterized by distinct ecological change. Anthropogenic climate change is increasing risk and frequency of natural disasters, with rising global temperatures leading to more devastating droughts, wildfires, and floods, as well as loss of life and agricultural capacity. The climate crisis is a systemic problem contributing to a multitude of socioeconomic, demographic, and political consequences (Kalwak and Weihgold, 2022) moving us toward what has been described as “Hothouse Earth” (McGuire, 2022), a phenomenon that cannot be reversed through human intervention once the tipping point is passed (Steffen et al., 2018). The Power Threat Meaning Framework (Johnstone et al., 2018) provides a lens through which different responses to climate breakdown including eco-distress, climate trauma and feelings of institutional betrayal may be understood. These are no longer issues that can be understood through traditional models for understanding psychological distress (e.g., the Diagnostic and Statistical Manual), but issues tied to wider contextual factors including vested interests of the fossil fuel industry, carbon intense lifestyles, geopolitics and war (Morgan et al., 2022). Developments in psychological science and ecophilosophy highlight an urgent need to foster a sense personal agency for the promotion of planetary wellbeing, rediscovering a sense of purpose and hope, and reconnecting with and cultivating compassion for the natural world, which will require reaching out to those with different personal values (Morgan et al., 2022; Pihkala, 2022). Despite the positive contributions of psychology, including the promotion of climate action (Gulliver et al., 2021), the field has been criticized for focusing on the individual rather than the system (Kern et al., 2020). Our own work (Kemp et al., 2017; Mead et al., 2019, 2021a; Kemp and Fisher, 2022; Wilkie et al., 2022), and the work of others (Kern et al., 2020; Lomas et al., 2021; Lambert et al., 2022), has highlighted how the combination of top-down (e.g., public policy) and bottom-up (i.e., individual behavior change) approaches may be combined to support responses to complex problems. Our focus in this paper is on the need for population-wide inner development and self-transformation to improve progress on the United Nations Sustainable Development Goals (UNSDGs), drawing on scientific developments embedded in existential and positive psychology.

## On the need for inner development for people and planet

The UNSDGs represent a universal call to action to achieve peace and prosperity for people and the planet by the year 2030 (https://sdgs.un.org/). These goals provide a blueprint for sustainability and focus on the promotion of good health and wellbeing, minimizing poverty, reducing inequalities, building sustainable cities and communities, and acting against climate change. Progress on the UNSDGs has been disappointing (see https://unstats.un.org/sdgs/report/2022/ for further information), indicating that policy mandates alone are not sufficient to spur collective action—we must *also* focus on individuals and communities at scale and nurture their capacities to promote planetary wellbeing. The suggestion that capacity for effective action will depend on the inner development and transformation of individuals (Wamsler and Brink, 2018; Woiwode et al., 2021) is an idea that has led to the so-called “inner development goals” (IDGs; https://www.innerdevelopmentgoals.org/).

The Inner Development Goals initiative highlights various skills and qualities for inner growth that must be supported in individuals, groups, and organizations if humanity is to achieve a sustainable global society in the face of complex societal and global issues. This includes cognitive and social skills, with a focus on being, thinking, relating, and civic engagement including collaboration and activism. These concepts are conterminous with developments in wellbeing science (Kern et al., 2020; Lomas et al., 2021; Mead et al., 2021a; Kemp and Edwards, 2022; Lambert et al., 2022), highlighting that actions to support planetary health and wellbeing are often synonymous to those required to achieve individual and collective wellbeing, highlighting the overlap between the complex constructs and systems of the modern world. Our own theoretical framework (the GENIAL model) focuses on similar concepts, including balanced minds, engaged communities, and connection to nature, around which we have promoted positive change at multiple levels of scale. We have defined the complex construct of wellbeing itself as a sense of connectedness to ourselves (the individual domain), others (the community domain), and nature (the environment domain) (Kemp et al., 2017; Mead et al., 2021b; Kemp and Fisher, 2022; Wilkie et al., 2022). Various societal crises reflect the result of a disconnection from ourselves, others, and nature (Bhaskar, 2012; Weintrobe et al., 2021); such that rebuilding our sense of connection (or “relatedness”, as per the Inner Development Goals) is crucial for supporting ourselves, communities and planet.

A sense of connection to the self (i.e., the individual) may be supported by activities which engage the body and mind, such as mindfulness. Defined as intentional, non-judgemental attention to the present moment (Kabat-Zinn, 2005), mindfulness has been linked to theories of attention and awareness (Brown et al., 2007; Sumantry and Stewart, 2021), with research demonstrating that regular mindfulness meditation influences structural changes in brain regions involved in learning, emotion regulation, self-referential processing and perspective taking (Hölzel et al., 2011). Mindfulness-based behavioral therapies, such as Acceptance and Commitment Therapy, focus on defusing thoughts, feelings, and experiences and explicitly seek to promote mental health and wellbeing by increasing meaningfulness and valued living (Hayes et al., 1999). Mindfulness may support clarification of one's values, with value-based living driving thoughtful future actions. Together these strategies offer one example of evidence-based and sustainable means for supporting inner development (Ericson et al., 2014) by broadening mindsets and increasing the capacity of individuals to deal with complex issues (such as climate change), reducing avoidance-based coping tendencies that may otherwise arise when overwhelmed (Centre for Research on Environmental Decisions, 2009). A focus on inner development may therefore encourage and facilitate “sustainability from within” (Wamsler et al., 2018).

We now provide examples of how we have sought to promote inner development within the context of education and the healthcare sector—two complex systems with great capacity to promote wellbeing at a population-wide level.

## Promoting inner development through complex systems

A system can be conceptualized as an integrated or interdependent set of elements forming a complex whole (WHO Regional Office for Europe, 2022). Systems-based approaches therefore emphasize a need for understanding dynamic interconnections between elements within a system (which may include individuals, populations, and organizations) to recognize how agents evolve in response to each other and their varying contexts (WHO Regional Office for Europe, 2022). Systems-informed thinking lends itself to addressing societal health challenges by taking a broader viewpoint which accounts for the complexity and interdependence of related and overlapping systems (Kreuter et al., 2004). Healthcare is one such system that is inherently complex (Tien and Goldschmidt-Clermont, 2009), as is the education sector. Recent efforts have combined developments in positive psychology with systems-based thinking to identify leverage points where meaningful change may occur (Kern et al., 2020). This approach has been successfully applied to education (Kern et al., 2020), with such developments inspiring our own work within the education and healthcare sectors (Kemp and Fisher, 2022). Our recent efforts have demonstrated how inner development may facilitate collective action aimed at societal challenges that include the climate crisis, demonstrated through the strategic design and delivery of an evidence-based module based on our GENIAL framework (Kemp et al., 2017; Mead et al., 2021a). This module educates students about the latest developments in wellbeing science and theory and empowers them to apply these ideas to promote individual, collective and planetary wellbeing (Kemp and Fisher, 2021; Kemp L. et al., 2022; Kemp A. H. et al., 2022). Delivery of this module was found to significantly improve levels of student wellbeing at a time of suffering and crisis—specifically during the height of the COVID-19 pandemic, during which time levels of anxiety and depression increased (Castaldelli-Maia et al., 2021) especially in those at a social or economic disadvantage (Gloster et al., 2020). It is this focus on building inner development alongside suffering that is a key part of second wave positive psychology (Wong, 2019).

Suffering is an inevitable feature of the human experience (Malpas and Lickiss, 2012; Wong, 2022). This is not to say that we must surrender to suffering, but rather that we must learn to transcend (Nhât Hạnh, 2014) and harness its potential to foster growth in contrast to experiential avoidance (Chawla and Ostafin, 2007). Existential positive psychology outlines the foundations for growth through adversity (Wong, 2019), with philosophical underpinnings based on the work of Frankl (1984), and more recently, the writings of Paul Wong (Wong, 2011, 2019, 2020). As evidenced by the COVID-19 pandemic, suffering can be a driver for positive change, ranging from small-scale changes at the individual level (such as health behaviors; Jaeger et al., 2021) to large-scale changes that impact upon the environment, such as reductions in pollution and greenhouse gas emission (Khan et al., 2021). However, change is not always permanent, as is evidenced by ever increasing emissions (Ripple et al., 2021; Davis et al., 2022) despite slight improvements during multi-national COVID-related lockdowns. Our own work has identified the role of tragic optimism (optimism despite suffering) in supporting wellbeing (Mead et al., 2021b) during the pandemic, along with routes through which post-traumatic growth can be achieved, including gratitude (a self-transcendent emotion) and connection to nature (Mead et al., 2022). Embedding these insights into the psychology curriculum and encouraging students to apply these principles to their own lives has the potential to scale up opportunities for positive change. We have also applied our GENIAL theoretical framework to our work within the healthcare sector leading to previously unimagined interventions for individuals with pervasive impairments resulting from acquired brain injury (Tulip et al., 2020; Wilkie et al., 2021; Gibbs et al., 2022a). This work is focused on achieving positive change at multiple levels of scale including the individual (e.g., post-traumatic growth), community (group-focused positive psychotherapy) and the organization (e.g., co-production and partnership working), positioning the individual within increasing phenomenological scales that extend to the ecosystem and life course, with important implications for the sustainability of the healthcare sector (Gibbs et al., 2022b).

## Laying the foundations for the Symbiocene

The Sustainable Development Goals and Inner Development Goals highlight the role that wellbeing and psychological science can play in securing a better future for ourselves and the planet. Small steps have been taken in positive psychology to move from a sole focus on the individual to include a focus on groups and societies (Lomas et al., 2021); we further these steps by highlighting the need for fostering inner development (a focus on the individual) in order to drive societal change supported by top-down initiatives (a focus on the community and environment) at a higher level through, for example, wellbeing public policy (e.g., Fabian and Pykett, 2022). Strategies which nourish inner dimensions and foster connection, community, and a belief in something greater than oneself have been described as an emerging “*recovery movement”* in response to the various crises we face (Koger, 2015). These crises in part, stem from an extreme disconnection from the self, others and nature (Bhaskar, 2012; Way et al., 2018; Weintrobe et al., 2021), and we argue therefore that methods to facilitate these connections may help to build our inner resources (Mead et al., 2021b; Wilkie et al., 2022), supporting individual change needed to achieve positive change at higher levels of scale. A focus on the inter-relationships of the self, others and nature will lay strong foundations for a new era described as the Symbiocene (from the Greek “sumbiosis”, or companionship; Albrecht and van Horn, 2016), in which all living beings live together harmoniously in mutual benefit, providing a potential antidote to the “*long emergency”* (Kunstler, 2007) of the climate crisis.

## Conclusions

We suggest that the health and wellbeing of individuals, communities and nature is dependent upon humanity moving toward a new epoch—the Symbiocene—characterized by an interconnectedness and “eco-homeostasis” between all living beings (Albrecht and van Horn, 2016). Progress on inner development must be supported alongside commitments to systemic change for a new “ecological economics” of a future post-growth society (Jackson, 2016). Inner development will play a key role in driving positive planetary change, and psychological scientists have a unique opportunity to facilitate such change by promoting the need for self-development and transformation to manage, cope and inevitably flourish despite suffering. This potential has motivated the development and continued refinement of our own GENIAL model, research and applications, guided by a need to better align sustainability and wellbeing agendas (Kemp et al., 2017; Mead et al., 2021b; Kemp and Fisher, 2022). The emerging fusion of ideas between sustainability literature, wellbeing science, and behavior change, offers huge potential for developing novel, evidenced-based approaches to societal transformation.

## Author contributions

JM and AK developed the manuscript aims and refined iterations of the manuscript. JM and KG developed the first iteration of the manuscript and integrated feedback into the manuscript. AK and ZF provided continuous feedback on variations of the manuscript. All authors contributed to the article and approved the submitted version.
